# A striatal-enriched intronic GPCR modulates huntingtin levels and toxicity

**DOI:** 10.7554/eLife.05449

**Published:** 2015-03-04

**Authors:** Yuwei Yao, Xiaotian Cui, Ismael Al-Ramahi, Xiaoli Sun, Bo Li, Jiapeng Hou, Marian Difiglia, James Palacino, Zhi-Ying Wu, Lixiang Ma, Juan Botas, Boxun Lu

**Affiliations:** 1 State Key Laboratory of Genetic Engineering, Department of Biophysics, School of Life Sciences, Fudan University, Shanghai, China; 2 Collaborative Innovation Center for Brain Science, Shanghai, China; 3 Department of Molecular and Human Genetics, Baylor College of Medicine, Houston, United States; 4 MassGeneral Institute for Neurodegenerative Diseases, Massachusetts General Hospital, Boston, United States; 5 Developmental Molecular Pathways, Novartis Institutes for Biomedical Research, Cambridge, United States; 6 Department of Neurology and Research Center of Neurology, Second Affiliated Hospital, School of Medicine, Zhejiang University, Hangzhou, China; 7 Department of Anatomy, Histology and Embryology, Shanghai Medical College, Fudan University, Shanghai, China; Trinity College Dublin, Ireland

**Keywords:** polyQ, neurodegeneration, GPCR, huntington's disease, cyclic AMP, intron, *D. melanogaster*, human, mouse

## Abstract

Huntington's disease (HD) represents an important model for neurodegenerative disorders and proteinopathies. It is mainly caused by cytotoxicity of the mutant huntingtin protein (Htt) with an expanded polyQ stretch. While Htt is ubiquitously expressed, HD is characterized by selective neurodegeneration of the striatum. Here we report a striatal-enriched orphan G protein-coupled receptor(GPCR) Gpr52 as a stabilizer of Htt in vitro and in vivo. Gpr52 modulates Htt via cAMP-dependent but PKA independent mechanisms. *Gpr52* is located within an intron of *Rabgap1l*, which exhibits epistatic effects on Gpr52-mediated modulation of Htt levels by inhibiting its substrate *Rab39B*, which co-localizes with Htt and translocates Htt to the endoplasmic reticulum. Finally, reducing Gpr52 suppresses HD phenotypes in both patient iPS-derived neurons and in vivo *Drosophila* HD models. Thus, our discovery reveals modulation of Htt levels by a striatal-enriched GPCR via its GPCR function, providing insights into the selective neurodegeneration and potential treatment strategies.

**DOI:** http://dx.doi.org/10.7554/eLife.05449.001

## eLife digest

Huntington's disease is an inherited disorder of the central nervous system. Symptoms typically begin between the ages of 30 and 50, and initially include clumsiness and uncontrollable movements, as well as personality changes and mood swings. Symptoms worsen over time and life expectancy is usually around 10 to 25 years following diagnosis.

The disease is caused by a mutation in the ‘*huntingtin*’ gene, which leads to the production of an abnormal form of ‘huntingtin’ protein. This accumulates inside neurons in a region of the brain called the striatum, which is involved in the control of movement, and destroys them. However, it is not clear why other regions of the brain that also produce the mutant huntingtin protein are not affected.

Yao, Cui, Al-Ramahi, Sun et al. have now identified a protein that could explain this phenomenon and open up new therapeutic possibilities for Huntington's disease. The protein, which is called Gpr52, is a receptor located within the outer membrane of neurons, particularly those in the striatum. Reducing the levels of this protein reduced the amount of mutant huntingtin protein that was able to accumulate inside cells grown in culture. Moreover, mice that were genetically engineered to possess a mutant *huntingtin* gene, but only a single copy of the gene for Gpr52, accumulated less mutant huntingtin in the striatum than mice with two copies of the Gpr52 gene.

Further experiments revealed that Gpr52 protects mutant huntingtin from being broken down inside cells: it does this by activating a signaling pathway involving the cellular messenger cAMP. Encouragingly, when genetic techniques were used to reduce Gpr52 synthesis in a fruit fly model of Huntington's disease, the treated flies showed fewer movement impairments than flies that had not been treated. In addition, reduced levels of Gpr52 were observed to lead to dramatic protective effects in neurons derived from the stem cells of a patient with Huntington's disease.

The fact that Gpr52 is located on the surface of neurons means that it might be possible to design drugs that can block its activity and thus reduce accumulation of mutant huntingtin. Such a treatment would be the first to target the causal mechanism behind Huntington's disease, rather than simply addressing the symptoms. The strategy could also be relevant to Alzheimer's disease, Parkinson's disease and other neurodegenerative disorders in which death of neurons is triggered by abnormal accumulation or aggregation of proteins.

**DOI:** http://dx.doi.org/10.7554/eLife.05449.002

## Introduction

Neurodegenerative disorders refer to a number of diseases caused by progressive loss of neurons, and they currently have no cure. Many similarities appear in these diseases, such as selective loss of neurons in certain brain regions and accumulation of aggregation-prone proteins (Soto, 2003). In order to study these fundamental features and find treatment strategies of these diseases, Huntington's disease (HD) is often used as an important model because of its clear genetics (The Huntington's Disease Collaborative Research Group, 1993), which facilitates establishment of genetic models as well as early diagnosis. The major cause of HD is the cytotoxicity of the mutant Htt protein (mHtt) (Rubinsztein and Carmichael, 2003), which is expressed throughout the brain and peripheral tissues, but elicits selective neurodegeneration of the corpus striatum and lesser damage to the cerebral cortex in HD patients (Cowan and Raymond, 2006). This selectivity is likely contributed, at least partially, by striatal-enriched modulators of mHtt toxicity and stability (Subramaniam et al., 2009; Tsvetkov et al., 2013). Consistent with this idea, the neuronal longevity correlates with mHtt turnover, which is slower in striatal than in cortical neurons (Tsvetkov et al., 2013), suggesting expression of striatal-enriched mHtt stabilizers. Discovery of such stabilizers may help understanding the selective pathology of HD.

More importantly, it provides potential therapeutic entry points for HD: while the mechanism of mHtt toxicity is unclear, lowering its level should suppress its downstream toxicity and treat the disease (Yu et al., 2014). Meanwhile, reducing the wild-type Htt protein (wtHtt) at the same time seems to be well-tolerated (Boudreau et al., 2009; Grondin et al., 2012; Lu and Palacino, 2013). Thus, modulators of Htt levels are attractive targets for potential HD treatment.

## Results

### Gpr52 modulates Htt levels in the striatal cells in vitro and in vivo

To identify modulators of Htt levels in the striatal cells, we screened through a number of candidates in STHdh^Q7/Q111^ cells, a well-established and easily-transfectable striatal-derived cellular HD model expressing endogenous full length mHtt (Trettel et al., 2000). We tested the endogenous mHtt levels following knock-down of 104 candidate modulators using pooled siRNAs. We selected these candidates based on our previous screening results in the stably-transfected *Drosophila* S2 cells (Lu et al., 2013) and tested the mHtt level changes by western-blots (Figure 1—figure supplement 1). This effort revealed six potential modulators of mHtt levels: Gpr52 and Eaf1 siRNAs lower mHtt, whereas Gclc, Grid2, Ndrg3 and Hdhd3 siRNAs increase its level (Figure 1—figure supplement 1).

Among them, Gpr52 (a GPCR) is of special interest. First, GPCRs locate on the plasma membrane and their functions are modulated by extracellular molecules, placing them among the most druggable targets: highly accessible to drugs and the functions are modulated by small molecules. Second, Gpr52 has been recently characterized as a Gα_s_-coupled receptor highly enriched in the striatum, especially D2 neurons (Sawzdargo et al., 1999; Komatsu et al., 2014), which are amongst the earliest affected in HD (Raymond et al., 2011). The coincidence between Gpr52 expression and selective neurodegeneration suggests that Gpr52 may contribute to the selective early loss of striatal neurons in HD.

**Figure 1.**
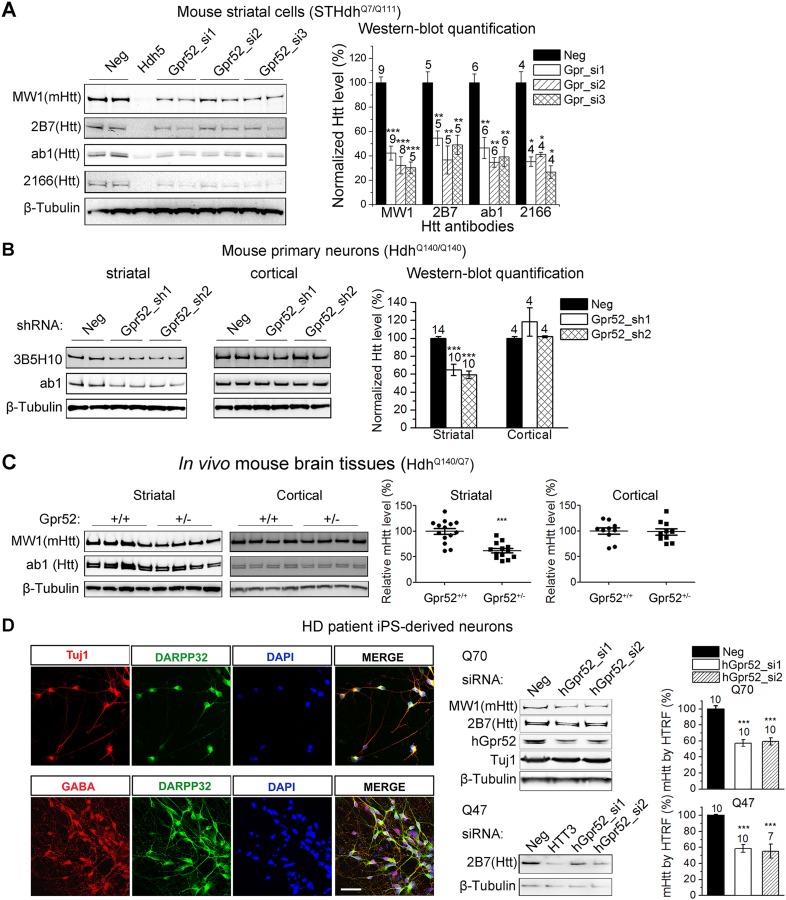
Gpr52 modulates Htt levels. All data plots: average and S.E.M.; ‘*’: p < 0.05, ‘**’: p < 0.01, ‘***’: p < 0.001 by the two-tailed Mann–Whitney U test. The number on top of each bar indicates the biological replicate number. (**A**) Transfection of Gpr52 siRNAs (Gpr52_si1∼3) in the mouse striatal cells (STHdh^Q7/Q111^) lowers Htt levels, as detected by Htt antibodies MW1, 2166, ab1 and 2B7. MW1 is the polyQ antibody that detects only the mHtt protein, whereas 2166, 2B7 and ab1 detects both mHtt and wtHtt. *Left* panels: representative western-blots; Hdh5 is the Htt siRNA used as the positive control for Htt knock-down. Neg is the non-targeting siRNA used as the negative control. *Right* panel: western-blot quantification from multiple replicates. (**B**) Infection of lentiviruses expressing Gpr52 shRNAs (Gpr52_sh1∼2) lowers Htt in primary striatal but not cortical neurons cultured from Hdh^Q140/Q140^ knock-in mice. *Left* panels: representative western-blots. *Right* panel: western-blot quantification for the normalized 3B5H10 signals from multiple replicates. (**C**) Heterozygous knockout of Gpr52 lowers Htt in vivo in the striata but not cortices of Hdh^Q140/Q7^ knock-in mice in vivo. The mice were obtained by crossing the heterozygous Gpr52 knockout mice with the Hdh^Q140/Q140^ knock-in mice. Littermates between 40 to 69 days of age were analyzed. *Left* panels: representative western-blots. *Right* panel: western-blot quantification of the normalized MW1 signals from multiple mouse samples. Each dot represents the signal from a single mouse. (**D**) *Left* panels: Immunostaining of HD patient iPS-derived striatal-like neurons. Differentiated neurons from HD patient's iPS cells express molecular markers for striatal medium spiny neurons: Tuj1, GABA and DARPP32. *Scale* bar: 50 μM. *Right* panels: Transfection of human Gpr52 siRNAs (hGpr52_si1∼2) in the HD patient iPS-derived neurons lowers Htt levels detected by both western-blots and HTRF. HTT3 is the Htt siRNA used as the positive control for Htt knock-down. Bar plot represents the normalized mHtt levels detected by HTRF using the 2B7/MW1 antibody pair. **DOI:** http://dx.doi.org/10.7554/eLife.05449.003

To confirm Gpr52's effect on Htt, we tested an additional set of siRNAs (Gpr52_si1∼3) in the STHdh^Q7/Q111^ cells, and observed robust reduction of both wild-type and mutant endogenous Htt levels (Figure 1A). Consistently, in the Hdh^Q140/Q140^ knock-in mice (Menalled et al., 2003), shRNA mediated knock-down of Gpr52 lowers Htt in primary cultured striatal but not the cortical neurons (Figure 1B). More importantly, by crossing the Gpr52 heterozygous knockout mice with the Hdh^Q140/Q140^ knock-in mice, we have observed robust lowering of endogenous Htt levels in the striata but not in the cortices by heterozygous knockout of Gpr52 (Figure 1C); confirming Gpr52 mediated modulation of Htt levels in vivo.

**Figure 1—figure supplement 1.**
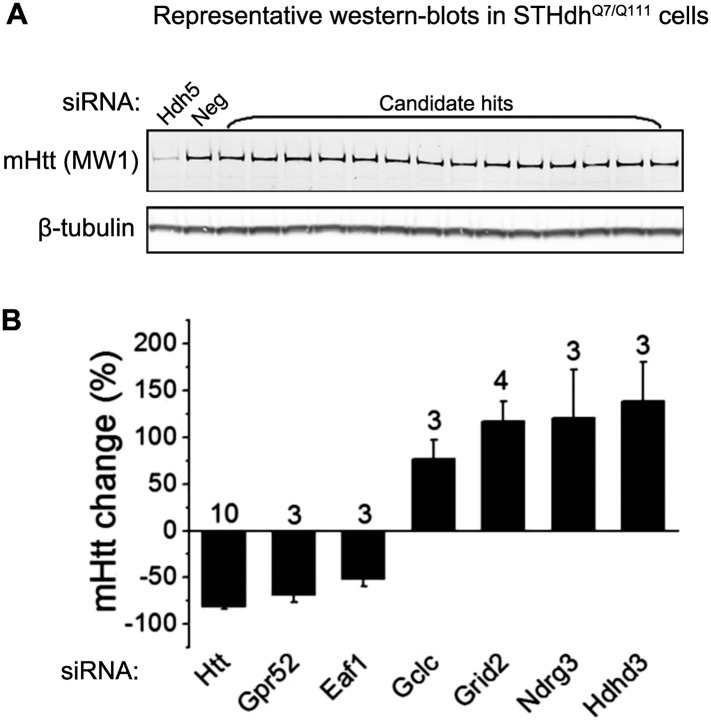
Screening for modulators of Htt levels in the striatal cells (STHdh). (**A**) Representative western-blots in the STHdh^Q7/Q111^ cells of candidate modifiers of mHtt levels. 104 candidate modifiers were selected based on our previous screening results (doi: 10.1038/nn.3367). The genes that had averaged Z score values larger than 1.2 and were not previously identified as validated hits in the patient fibroblasts were selected for testing in STHdh cells by pooled siRNAs (Dharmacon, custom library). The MW1 detected full length mHtt is quantified and normalized to the tubulin signal using the ImageJ software. The siRNAs that change mHtt levels by more than 30% in the same direction in three biological repeats were selected as hits. The number on top of each bar indicates the biological repeat number of siRNA knock-down. (**B**) The bar plot of mHtt level changes upon knock-down of the identified modifiers. The numbers on top of each bar represent the number of biological replicates tested. **DOI:** http://dx.doi.org/10.7554/eLife.05449.004

**Figure 2.**
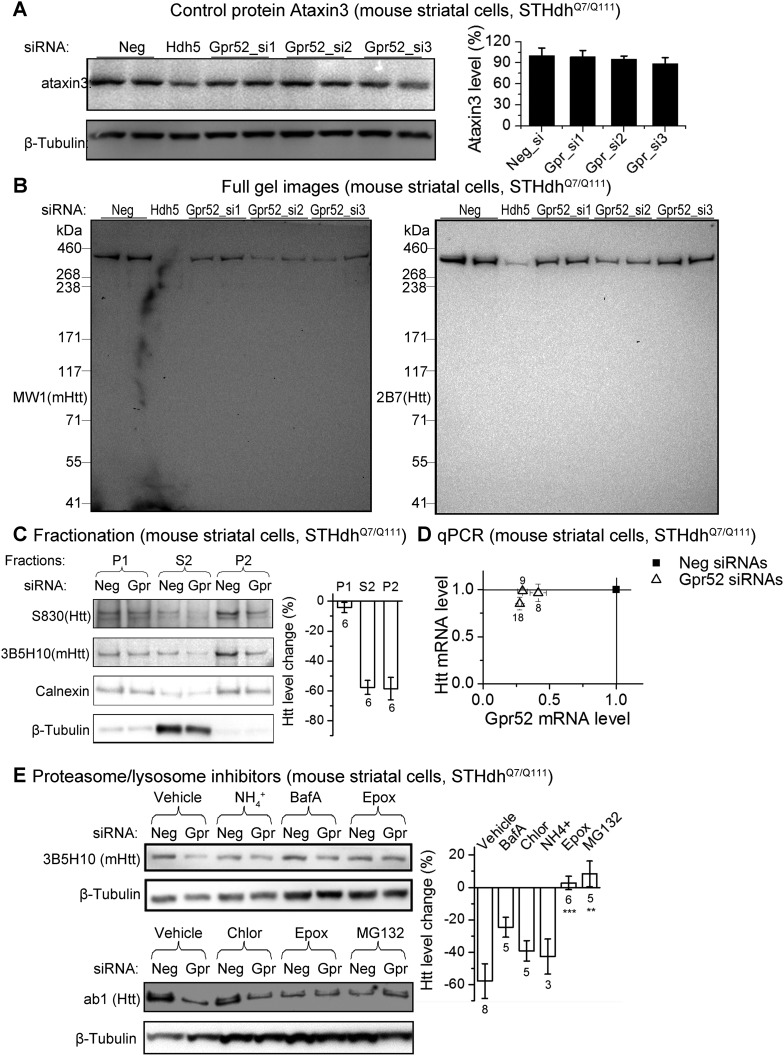
The Gpr52 mediated change of Htt levels is via protein degradation. (**A**) Representative western-blots of STHdh^Q7/Q111^ cell lysates showing no reduction of Ataxin3 levels by Gpr52 knock-down. *Bar* graph: quantification of Atxn3 levels, n = 4. (**B**) Full membrane images of Htt blots showing no increase/appearance of lower molecular weight bands in the STHdh cell lysates, suggesting that the Htt reduction by Gpr52 knock-down is not due to protein cleavage modulations. (**C**) Representative western-blots for different biochemical fractions of the protein extract from STHdh^Q7/Q111^ cells transfected with non-targeting control siRNA (Neg) or the Gpr52 siRNA (Gpr52_si2). Gpr52 knock-down reduced Htt levels in both the P2 and S2 fractions, but not the P1 fraction. (**D**) In the Gpr52 siRNAs (triangles) transfected STHdh^Q7/Q111^ cells, Htt mRNA levels (Y-axis) and Gpr52 mRNA (X-axis) levels were measured by qPCR. Both the Gpr52 siRNAs and the Htt siRNAs show substantial knock-down of their targets, whereas the Gpr52 knock-down by Gpr52 siRNAs do not reduce Htt mRNA levels. No reverse-transcriptase control samples have been assayed to eliminate potential contaminations from genomic DNA. (**E**) *Left*: representative western-blots of STHdh^Q7/Q111^ cells transfected with non-targeting control siRNA (Neg) or the Gpr52 siRNA (Gpr52_si2) with or without proteasome or autophagy inhibitors. *Right*: the bar plot of western-blot quantification of the Htt level change by Gpr52 siRNA transfection with each compound treatment. **DOI:** http://dx.doi.org/10.7554/eLife.05449.005

To test Gpr52's effect in human neurons, we generated HD patient iPS-derived neurons mimicking the striatal medium spiny neurons (Figure 1D). These cells exhibit neuronal morphology with a high percentage expressing the neuronal marker Tuj1, as well as the medium spiny neuronal marker Darpp32 (Ouimet et al., 1984) and the neurotransmitter GABA (Figure 1D). Because HD patient striatal neurons are unavailable for culture, these neurons represent their closest culture model. Consistently, detected by both western-blots and Homogenous Time-Resolved Förster Resonance Energy Transfer (HTRF) assays (Weiss et al., 2009), knocking-down Gpr52 significantly reduces Htt in these cells (Figure 1D). The effect is relatively specific, because levels of loading control proteins (Figures 1, 2) and another polyQ protein ataxin3 (Figure 2A) remain unchanged. The Htt lowering is not caused by antibody binding artifacts or Htt cleavage, as multiple antibodies detect comparable reduction (Figures 1, 2) and there is no obvious increase of possible Htt fragments of lower molecular weights that may account for the lowering of the full length protein (Figure 2B). We further tested different biochemical fractions of the lysates including P1, P2 and S2, representing the crude nuclear fraction, the membrane/organelle fraction, and the cytosolic soluble fractions, respectively (Kegel et al., 2005). The major Htt lowering occurs in both the P2 and S2 fractions, but not the P1 fraction (Figure 2C). In addition, the Htt mRNA level is not affected by Gpr52 (Figure 2D), and the Gpr52's effect is completely blocked by treatment with proteasome inhibitors (Figure 2E), suggesting that the modulation is mainly mediated by proteasomal degradation.

### Gpr52 mediated modulation of Htt levels is cAMP-dependent but PKA-independent

**Figure 3.**
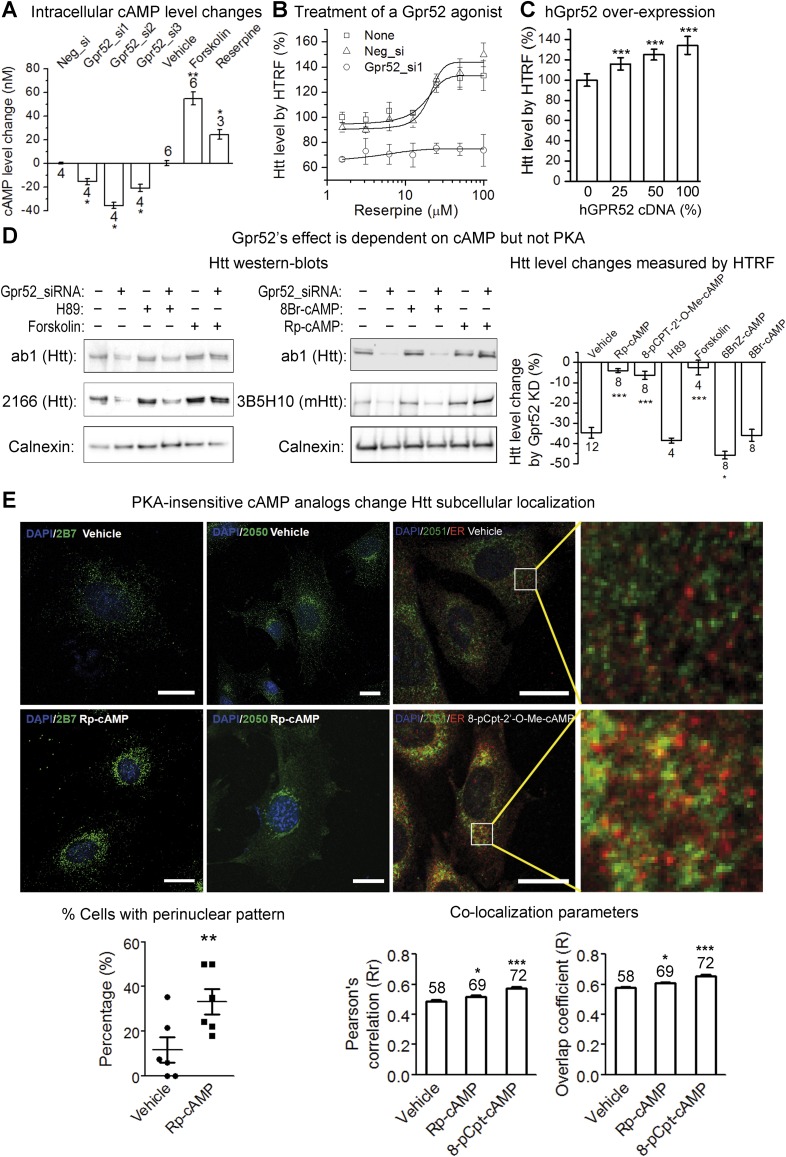
Gpr52 modulates Htt levels via cAMP dependent but PKA independent pathways. All experiments are performed in the mouse striatal cell line STHdh^Q7/Q111^, and all data are plotted as average and S.E.M. ‘*’: p < 0.05, ‘**’: p < 0.01, ‘***’: p < 0.001. The number on top of each bar indicates the biological replicate number. (**A**) Changes of cAMP levels measured by the cAMP-Glo assay (Promega). Gpr52 siRNAs were tranfected for 3 days, whereas the compound treatment (forskolin: 1 µM; reserpine: 10 µM) lasts for 24 hr; statistical analyses performed by the two-tailed Mann–Whitney U test. (**B**) Htt level measured by the 2B7/2166 HTRF (Liang et al., 2014) upon treatment of different doses of the Gpr52 agonist reserpine for 48 hr, with transfection of Gpr52 siRNA (Gpr52_si1) vs the non-targeting control (Neg_si), n = 4. (**C**) Htt level measured by the 2B7/2166 HTRF when transfected with hGPR52 cDNA titrated with the empty control vector at different percentages (X-axis), n = 6; statistical analysis performed by the one-way ANOVA and post-hoc Dunnett's test. (**D**) *Left and middle*: Representative western-blots of STHdh^Q7/Q111^ cells transfected with the Gpr52 siRNA (Gpr52_si2) and then treated with the indicated PKA modulators or cAMP analogs (forskolin and cAMP analogs: 1 µM; H89: 50 µM). Calnexin has been used as a loading control. *The Bars plot*: Htt level changes (%) measured by 2B7/2166 HTRF (Liang et al., 2014) of the total lysates with the same treatments as in the western-blot samples. (**E**) Confocal microscopy experiments showing that HTT proteins are enriched in the perinuclear and co-localize with the endoplasmic reticulum (ER) marker calreticulin upon treatment of Rp-cAMP or 8-pCpt-2′-O-Me-cAMP (8-pCpt-cAMP for short). *Upper panels*: representative images showed the immunofluorescent signals of Htt (green), ER marker (red, only in the third and fourth columns) and DAPI (blue) in STHdh Q7/Q111 cells treated by vehicle, 1 μM Rp-cAMP or 1 μM 8-cpt-cAMP. *Scale bars*, 20 μM. The two panels on the right side are magnified images from the left for visualizing the co-localization. Yellow pixels indicate co-localization. *Lower left plot*: the percentage of cells showing clear perinuclear pattern in each samples. The pattern was judged blindly. *Lower middle and right plots*: co-localization parameters including Pearson's coefficient and overlap coefficient (mean and S.E.M.). Numbers in indicate the number of cells analyzed for each treatment from five or more biological replicates. **DOI:** http://dx.doi.org/10.7554/eLife.05449.006

Gpr52 is enriched in the striatum (Sawzdargo et al., 1999; Komatsu et al., 2014), and thus may contribute to the selective stabilization of Htt there (Tsvetkov et al., 2013). Consistent with this, over-expression of Gpr52 cDNA (Figure 3—figure supplement 1) or treatment with an agonist reserpine (Komatsu et al., 2014) (Figure 3A) leads to a dose-dependent increase of Htt (Figure 3B–C), confirming Gpr52 as an Htt stabilizer.

**Figure 3—figure supplement 1.**
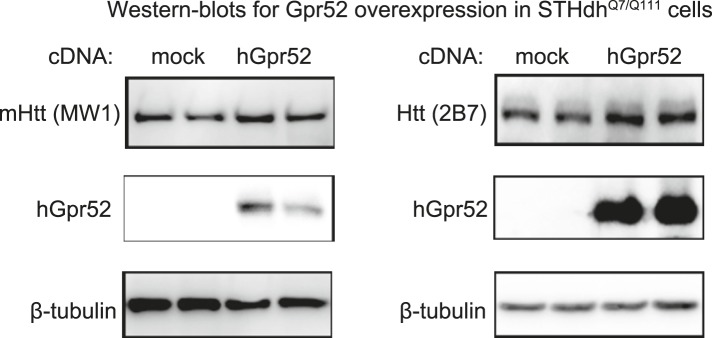
Over-expression of human Gpr52 cDNA in STHdh cells. Representative western-blots showing increased Htt levels and over-expression of human Gpr52 (hGpr52) in STHdh^Q7/Q111^ transfected with the hGpr52 cDNA. **DOI:** http://dx.doi.org/10.7554/eLife.05449.007

**Figure 3—figure supplement 2.**
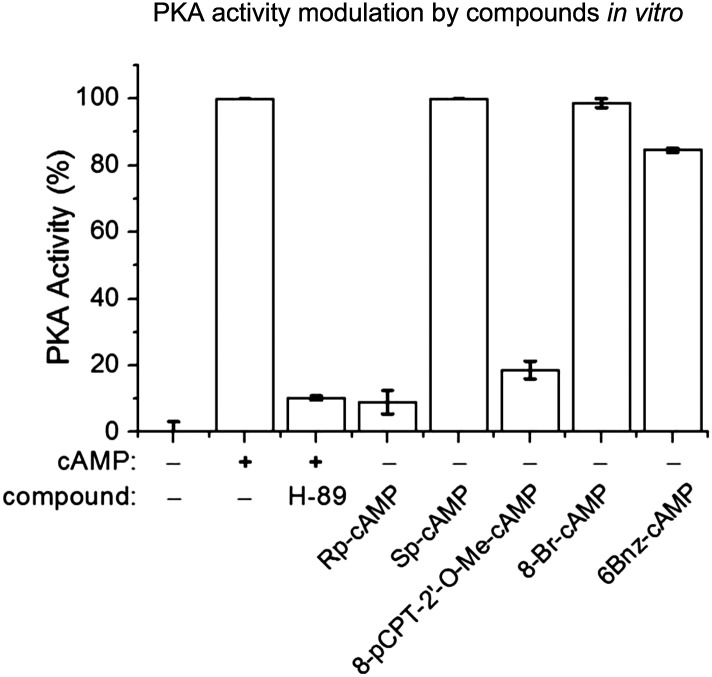
Modulation of PKA activity by PKA inhibitor or cAMP analogs. PKA kinase activities are measured by the cAMP-Glo kit (Promega) with a modified protocol in the *in vitro* condition. Basically, PKA activity is determined by the phosphorylation of the kinase substrate, which is detected by the reduction of the luciferase signal after 1 hr. The average signal without any cAMP or analogs is used as the baseline (0% PKA activity, bar 1). The average signal with 200 nM purified cAMP (a component in the kit) is set as the 100% PKA activity. The PKA inhibitor H89 was applied together with 200 nM cAMP and blocked ∼90% of the PKA activity. Different cAMP analogs (200 nM) are tested for their ability to activate PKA. n = 6 for each sample. Rp-cAMP and 8-pCPT-2′-O-Me-cAMP do not activate PKA, whereas Sp-cAMP, 8-Br-cAMP and 6Bn-cAMP activate PKA as efficiently as cAMP. **DOI:** http://dx.doi.org/10.7554/eLife.05449.008

**Figure 3—figure supplement 3.**
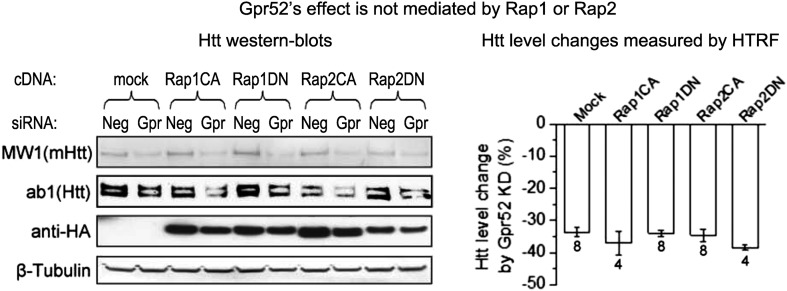
Gpr52's effect is not mediated by Rap1 or Rap2. *Left:* Representative western-blots of STHdh^Q7/Q111^ cells transfected with the Gpr52 siRNA (Gpr52_si2) versus control siRNA, and then with constitutively active or dominant negative Rap1 (Rap1CA or Rap1DN) or Rap2 (Rap2CA or Rap2DN). The Bars plot: Htt level reduction (%) measured by 2B7/2166 HTRF of the total lysates of cells with same transfections as in the western-blots. **DOI:** http://dx.doi.org/10.7554/eLife.05449.009

As a Gα_s_-coupled receptor, Gpr52 increases intracellular cyclic adenosine monophosphate (cAMP) levels when activated (Komatsu et al., 2014). Consistent with this, the intracellular cAMP level is reduced by knocking-down Gpr52 (by 15.6 ± 2.5 nM, 35.8 ± 2.5 nM and 21.1 ± 3.1 nM, respectively; Figure 3A), suggesting some levels of constitutive activity. Uncovering the possible involvement of this cAMP reduction in lowering Htt is critical, because it determines whether we can target Gpr52's GPCR function to lower Htt. Thus, we tested the effect of the adenylyl cyclase activator forskolin, which increases intracellular cAMP levels (by 54.8 ± 5.7 nM; Figure 3A). Treatment of forskolin completely inhibits the Htt reduction caused by the Gpr52 knock-down (Figure 3D, *left* panel, lane 5 and 6 compared to lane 1 and 2; *Bar plot*), suggesting that the reduction of cAMP is required for the Gpr52 mediated Htt modulation. The canonical cAMP sensor is protein kinase A (PKA) (Meinkoth et al., 1993). To test possible PKA-dependence, we treated the cells with Rp-cAMP, a non-hydrolysable cAMP analog that does not activate PKA (Rothermel and Parker Botelho, 1988) (Figure 3—figure supplement 2). Rp-cAMP completely inhibits the Gpr52's effect on Htt levels (Figure 3D, *middle* panel, lane 5 and 6 compared to lane 1 and 2; *bar plot*), indicating that the Htt reduction by knocking-down Gpr52 is mediated via a PKA-independent sensor of cAMP levels. Note that Rp-cAMP was applied at 1 µM, which is much lower than the *K*_i_ value for Rp-cAMP's inhibition of PKA (Rothermel and Parker Botelho, 1988) and thus should not influence the PKA activity. Similarly, another cAMP analog 8-pCPT-2′-O-Me-cAMP that does not activate PKA (Enserink et al., 2002) (Figure 3—figure supplement 2) completely abolishes the Htt reduction (Figure 3D; *Bar plot*) as well. In contrast, the PKA inhibitor H89 (Takuma and Ichida, 1994) (Figure 3—figure supplement 2) and a PKA-specific agonist 6-Bnz-cAMP (Christensen et al., 2003) fail to inhibit the Htt reduction by Gpr52 knock-down (Figure 3D), confirming the PKA-independence. Finally, the hydrolysable cAMP analog 8-Br-cAMP has little effect on the Gpr52 mediated Htt reduction (Figure 3D, *middle* panel, lane 3 and 4 compared to lane 1 and 2; *bar plot*), whereas the non-hydrolysable cAMP analogs 8-pCPT-2′-O-Me-cAMP and Rp-cAMP block the Gpr52's effect entirely (Figure 3D), indicating that a sustained increase of the cAMP level is needed to abolish the Gpr52's effect.

Other than PKA, the guanine exchange factor (GEF) Epac, Rapgef2 and potentially other GEFs have been reported as cAMP sensors that mediate downstream signals (de Rooij et al., 1998; Emery and Eiden, 2012; Emery et al., 2013). Thus, Gpr52 may function via a PKA-independent and cAMP-dependent signaling mechanism by activating GEFs. The reported small GTPases downstream of these GEFs are Rap1 and Rap2, and thus we tested their potential involvement by dominant negative and constitutively active Rap proteins (Fu et al., 2007). None of these showed obvious effects on the Htt lowering by Gpr52 knock-down (Figure 3—figure supplement 3), suggesting novel mechanisms potentially involving other GEFs and/or small GTPases, which is suggested in other models as well (Emery and Eiden, 2012; Kuwayama et al., 2013). Given that the major function of small GTPases is trafficking, we tested potential Htt translocation events upon treatments of PKA-insensitive analogs Rp-cAMP or 8-pCPT-2′-O-Me cAMP, and found that they lead to Htt enrichment at the perinuclear regions and co-localization with the endoplasmic reticulumn (ER) marker (Figure 3E). Translocation of Htt to the ER may prevent its proteasomal degradation due to lack of proteasomes in the ER (Plemper et al., 1997).

### *Rabgap1l* contains *Gpr52* in its intron and shows epistatic effect on the Gpr52 mediated Htt changes

**Figure 4.**
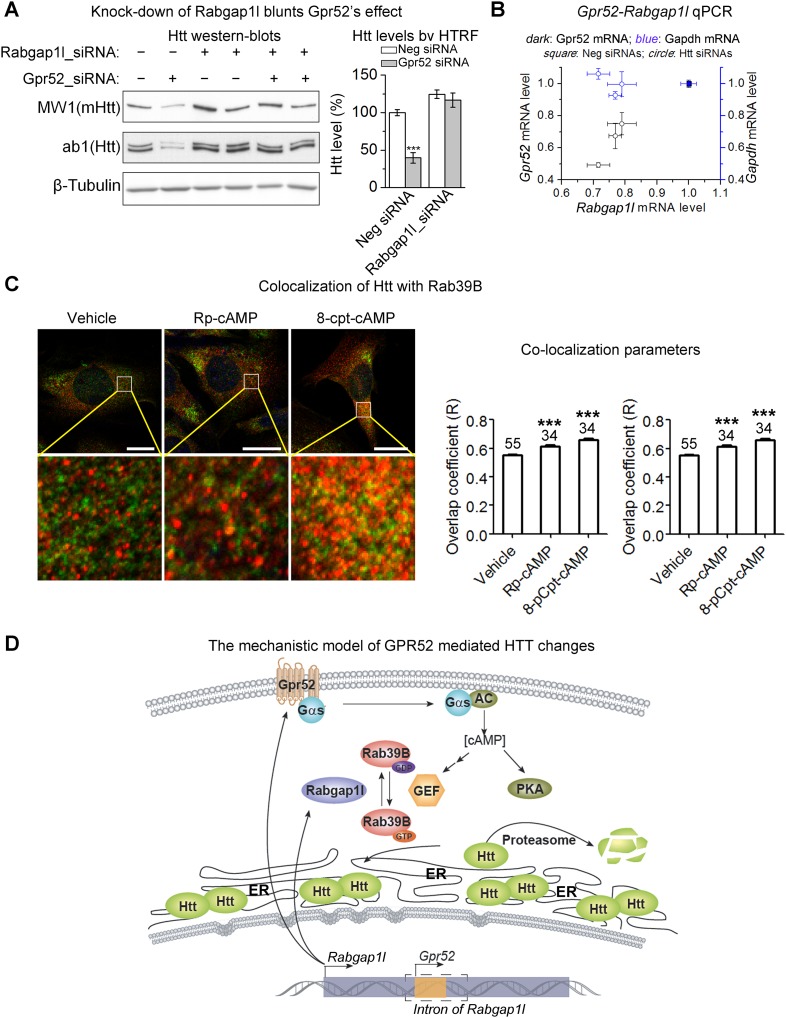
*Rabgap1l* interferes with Gpr52-mediated Htt modulation. (**A**) *Left*: Representative western-blots of STHdh^Q7/Q111^ cells transfected with the Gpr52 siRNA (Gpr52_si2) vs the control siRNA, along with or without the Rabgap1l siRNA. Rabgap1l knock-down blunts the Gpr52's effect on the Htt level. *Bar plot*: HTRF quantification of the transfected cells as indicated, showing consistent results with the western-blots; statistical analyses by the two-tailed Mann–Whitney U-test. (**B**) mRNA levels of *Gpr52* (*left* Y-axis) or Gapdh (*right* Y-axis) vs the ones of *Rabgap1l* (X-axis) upon transfection with HTT siRNAs or control (Neg). Both *Gpr52* and *Rabgap1l* mRNA levels are lowered upon HTT knock-down, and a correlation between the lowering is observed. (**C**) *Left panels*: Representative confocal microscopic immunofluorescent images of Htt and Rab39B in STHdh^Q7/Q111^ cells. Treatments with 1 μM RP-cAMP or 1 μM 8-pCPT-2′-O-Me-cAMP for 48 hr leads to increased co-localization between Htt and Rab39B. Red: Rab39B; Blue: DAPI; Green: Htt (using antibody 2051); The lower panels are magnified images from the left for visualizing the co-localization. Yellow pixels indicate co-localization. *Scale* bars: 20 μm. *Right panels*: co-localization parameters including Pearson's coefficient and overlap coefficient (mean and S.E.M.). Numbers in indicate the number of cells analyzed for each treatment from five or more biological replicates. (**D**) A model figure explaining the modulation of Htt levels by Gpr52. Gpr52 increases cAMP when activated, which leads to activation of an unknown GEF (Guanine Exchange Factor) that activates the downstream Rab39B protein. Rab39B then co-localize with Htt and translocate it to the ER, where the proteasomal degradation is prohibited due to lack of proteasomes in the ER. The *Gpr52* gene locates in an intron of the *Rabgap1l* gene, which expresses the GAP (GTPase Activating Protein) for Rab39B, and thus blocks the modulation. Thus, Gpr52 and Rabgap1l provide balanced regulation of Htt in striatal cells, and the shared genomic loci may facilitate their balance in modulating Htt levels via co-regulated expression in striatal cells. **DOI:** http://dx.doi.org/10.7554/eLife.05449.010

In the genomes of vertebrates including human, monkey, mouse, rat, dog, chicken and zebrafish, *Gpr52* is a single exon gene that is located in the intron of another gene *Rabgap1l*, which encodes a GTPase-activating protein (GAP) in the same orientation. *Gpr52* is located within the intron of the GAP domain-containing splice variants (GAP transcripts) only, but not the non-GAP transcripts (Figure 4—figure supplement 1A), suggesting possible functional links. GAPs function in opposition to GEFs in regulating small GTPase activities, and thus Rabgap1l may function to balance certain GEFs activated by Gpr52 via cAMP. The ‘co-localization’ of *Gpr52* and *Rabgap1l* in the genome might facilitate co-regulations of their expressions in certain cell types, so that they can balance each other's function in regulating Htt levels. Consistent with this hypothesis, knocking-down Rabgap1l increases Htt levels (Figure 4—figure supplement 1B) and blunts the Gpr52's effect (Figure 4A, Htt lowering drops from 60.3 ± 7.1% to 6.3 ± 7.7%). Interestingly, both *Gpr52* and *Rabgap1l* mRNA levels are lowered when HTT is knocked-down (Figure 4B, *dark* axis). As a control, *Gapdh* mRNA levels do not change (Figure 4B, *blue* axis). This suggests that *Gpr52* and *Rabgap1l* could be co-regulated together, possibly facilitated by their shared genomic locus, via possible feedback regulation by Htt. In summary, Gpr52 modulates Htt via a cAMP-dependent and PKA-independent signaling pathway, and its ‘host’ gene *Rabgap1l* exhibits epistatic effects on this modulation.

**Figure 4—figure supplement 1.**
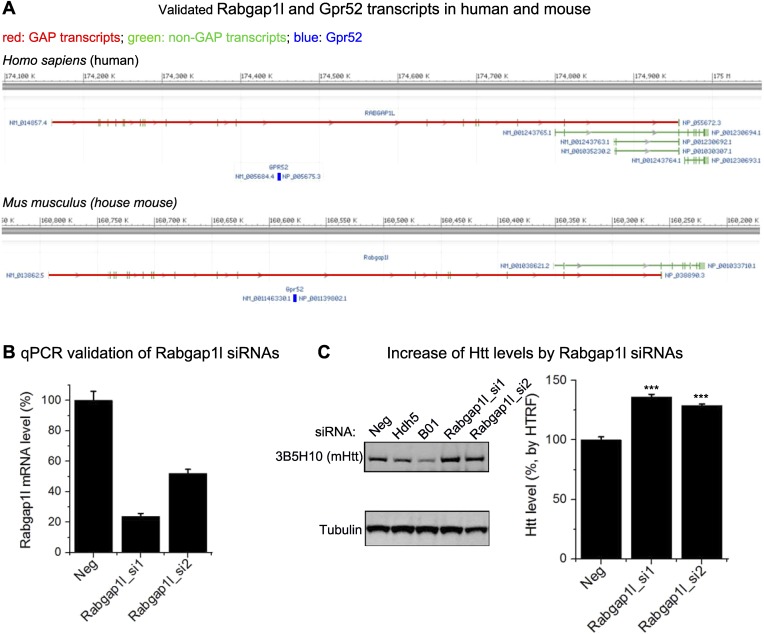
*Rabgap1l* genomic information and siRNA validation. (**A**) Genomic loci of Rabgap1l and Gpr52. (**B**) qPCR quantification of the Rabgap1l mRNA level of STHdh cells transfected with Rabgap1l siRNAs or the non-targeting control siRNA (Neg). 50–80% knock-down could be achieved by siRNA transfection in these cells. (**C**) Western-blot (*left*) and HTRF (*right*) experiments showing that Rabgap1l knock-down by siRNA increases the Htt level in the STHdh cells. Hdh5 and B01 are Htt siRNAs used as positive controls. For HTRF, the 2B7/2166 antibody pair was used. Data are plotted as mean and S.E.M, n = 16 for non-targeting siRNA control (Neg) samples, and n = 12 for Rabgap1l siRNA transfected samples. ‘***’: P < 0.001 by the two-tailed Mann–Whitney U-test. **DOI:** http://dx.doi.org/10.7554/eLife.05449.011

**Figure 4—figure supplement 2.**
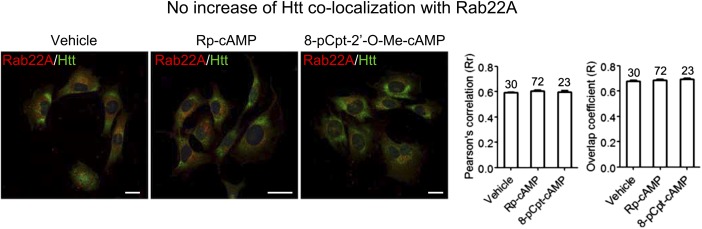
No increase of Htt co-localization with Rab22A. Representative confocal microscopic immunofluorescent images of Htt and Rab22A in STHdh^Q7/Q111^ cells and the quantification analysis of co-localization. Treatments with 1 μM RP-cAMP or 1 μM 8-pCPT-2′-O-Me-cAMP for 48 hr leads to no change of co-localization between Gpr52 and Rab22A. Red: Rab22A; Blue: DAPI; Green: Htt (using antibody 2051); *Scale* bars: 20 μm. **DOI:** http://dx.doi.org/10.7554/eLife.05449.012

**Figure 4—figure supplement 3.**
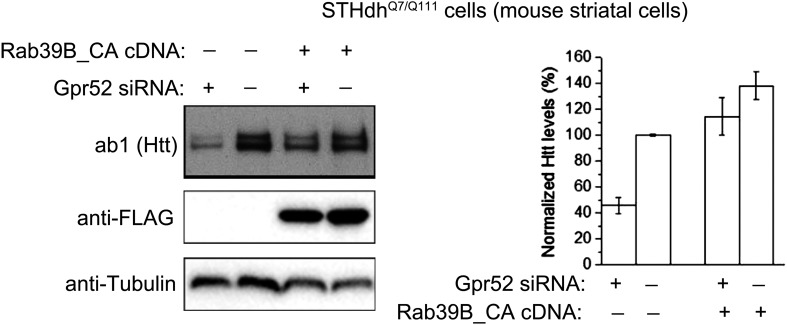
Constitutively active Rab39B blunts Gpr52's effect. *Left:* Representative western-blots of Htt (ab1), consitutively active Rab39B (anti-FLAG for Rab39B_CA), and the loading control (Tubulin). ‘−’ indicates the empty vector or scrambled siRNA controls. *Right:* quantification of the normalized Htt signals, n = 4. **DOI:** http://dx.doi.org/10.7554/eLife.05449.013

### The small GTPase Rab39B co-localizes with Htt and translocates Htt to the endoplasmic reticulumn when treated with cAMP analogs

The involvement of Rabgap1l gives the clue for identifying the downstream small GTPase(s) that mediates the modulation of Htt levels. There are three reported substrates for Rabgap1l: Rab22A, Rab39B and Rab34 (Itoh et al., 2006), among which Rab22A and Rab39B are expressed in the brain (http://www.biogps.org/; http://www.brain-map.org/). We thus tested whether Rab22A or Rab39B is the small GTPase downstream of Gpr52 and cAMP to mediate the Htt modulation.

Interestingly, Rab39B but not Rab22A shows significantly increased co-localization with Htt when the cells are treated with cAMP analogs Rp-cAMP or 8-pCPT-2′-O-Me cAMP (Figure 4C; Figure 4—figure supplement 2), suggesting that Rab39B may participate in the translocation of Htt to the ER. Consistent with this, expressing the constitutively active form of Rab39B blunts the Gpr52's effect (averaged Htt lowering reduces from 54.1 ± 6.3% to 17.2 ± 10.6%), confirming Rab39B's involvement in the pathway (Figure 4—figure supplement 3).

Taken together (Figure 4D), the presence of Gpr52 leads to sustained elevation of intracellular cAMP levels, which activates potential GEF(s) and subsequently its substrate Rab39B. Rab39B co-localizes with Htt and changes the localization of Htt, protecting it from degradation. Rab39B is inactivated by its GAP Rabgap1l, which shows epistatic effects on Gpr52 mediated Htt modulation. Gpr52 and Rabgap1l locate in the same genomic loci, which may facilitate their balanced regulation of Htt levels in the striatal cells.

### Targeting Gpr52 rescues neurons in HD models

**Figure 5.**
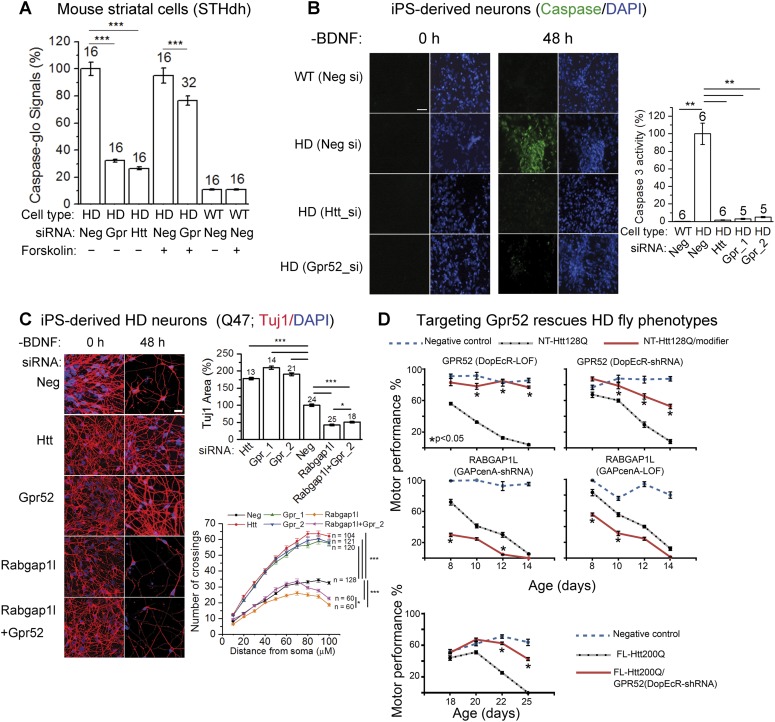
Lowering Gpr52 rescues human HD neurons and the in vivo fly HD models. (**A**) Caspase-glo of STHdh^Q7/Q111^ (HD) or STHdh^Q7/Q7^ cells (WT) with indicated transfections (Neg: the non-targeting controls siRNA; Gpr: the Gpr52 siRNA smartpool; Htt: the Htt siRNA Hdh5) and compound treatments; statistical analyses by the two-tailed Mann–Whitney U-test. (**B**) Caspase 3 activity of patient iPS-derived neurons (Q47) measured by the fluorescent indicator dye before and after BDNF removal (*scale* bar: 200 µm). Bar plots: quantification of caspase 3 signals corrected by the total cell number (by DAPI) and normalized to the HD controls (second bar). Statistical analyses were performed by the two-tailed Mann–Whitney U-test. (**C**) Immunostaining of Tuj1 and DAPI showing loss of neurons of HD patient iPS-derived neurons (Q47) cultured under the BDNF-deprived condition with indicated transfections, and the rescue by knocking-down Htt or Gpr52 or Rabgap1l or Rabgap1l + Gpr52 (*scale* bar: 200 µm). Bar plots: quantification of the area in each field covered by the Tuj1 signal (Tuj1 area) and the nuclei counts. All data normalized to the non-targeting siRNA transfected control samples. Statistical analyses were performed by the two-tailed Mann–Whitney U-test. Sholl plots: the sholl analysis (Sholl, 1953) results plotted for each sample, ‘n’ indicated the number of analyzed neurons. Statistical analysis performed by two-way ANOVA tests. (**D**) Age-dependent motor performance in normal flies expressing elav-GAL4 driver alone (Negative control, blue dotted lines), HD flies expressing elav-GAL4 driven NT-Htt128Q or FL-Htt200Q alone (black dotted lines), or HD flies crossed to loss of function mutation (LOF) or knock-down (shRNA) lines of Drosophila homologs of *Gpr52* or *Rabgap1l* (red lines). Lowering Gpr52 rescues the motor behavior deficits, whereas lowering Rabgap1l enhances the phenotype. n = 15, statistical analysis performed by one way ANOVA and Dunnett's post-tests. **DOI:** http://dx.doi.org/10.7554/eLife.05449.014

To test whether targeting Gpr52 may benefit HD neurons, we examined several HD-related phenotypes. STHdh^Q111/Q111^ cells exhibit mHtt-dependent caspase 3 and/or 7 activation by stress, such as serum removal (Miller et al., 2012; Lu et al., 2013). This provides readout for mHtt-dependent toxicity in striatal cells. Knocking-down Gpr52 remarkably reduces serum starvation-induced caspase 3 and/or 7 activity in STHdh^Q111/Q111^ cells (by 67.8 ± 1.1%; Figure 5A), indicating a suppression of the mHTT-induced toxicity. Treatment of forskolin substantially blunts this effect (to 17.3 ± 4.8%; Figure 5A), confirming that the Gpr52's effect is mainly mediated via the cAMP-dependent Htt reduction. Consistently, in the HD patient iPS-derived striatal neurons, knocking-down Gpr52 suppresses the mHtt-dependent neuronal loss and caspase 3 activation induced by withdrawn of the brain-derived neurotrophic factor (BDNF) (HD iPS Consortium, 2012; Lu and Palacino, 2013) (Figure 5B–C). In addition, knocking-down Rabgap1l exacerbate the neuronal loss and attenuates Gpr52's rescue (Figure 5C), confirming the involvement of Rabgap1l.

To validate the above observations in vivo, we tested the effect of modulating Gpr52 levels in a *Drosophila* model of HD expressing the N-terminal human Htt fragment with 128Q (Figure 5D, NT-Htt128Q) (Al-Ramahi et al., 2006). Decreased levels of *Drosophila* Gpr52 either by shRNA knockdown or by loss-of-function mutation (LOF) causes a robust suppression of the motor deficits induced by expression of this mHtt fragment in the *Drosophila* central nervous system (Figure 5D). Meanwhile, consistent with cellular models where Rabgap1l functions in opposition to the effect of Gpr52, knock-down of the *Drosophila* homolog of Rabgap1l exacerbates the HD motor deficits in the in vivo fly model (Figure 5D). The rescue effect by knocking-down Gpr52 is further validated in a *Drosophila* model of HD expressing the full length human Htt protein with 200Q (FL-Htt200Q). Expression of Gpr52 shRNA substantially improves the motor defects in those flies as well (Video 1). To test if Gpr52 knock-down rescue the HD cell survival in vivo, we tested the retina degeneration phenotype of the fly HD model and failed to observe a significant rescue (not shown), probably due to a lack of expression of Gpr52 in the retina and the supporting cells.

**Video 1.** The representative video showing that lowering Gpr52 rescues the in vivo fly full-length HD models–linked to Figure 5D, the lower panel. **DOI:** http://dx.doi.org/10.7554/eLife.05449.015

## Discussion

### Gpr52 as a striatal-enriched modulator of Htt levels

We have revealed Gpr52 as a striatal-enriched modulator of Htt levels. This provides a possible mechanism of selective neurodegeneration in the striatum, although other mechanisms may contribute as well; including striatal-enriched post-translational modifiers of Htt such as *Rhes* (Subramaniam et al., 2009), or non–cell-autonomous mechanisms (Wang et al., 2014). Gpr52 is enriched in the D2 neurons (Komatsu et al., 2014), which are amongst the earliest affected in HD (Raymond et al., 2011), possibly reflecting their susceptibility to mHtt due to expression of Gpr52. Further evidence needs to be obtained to reveal whether Gpr52 is one of the major causes of the regional specificity. For example, Gpr52 could be exogenously expressed in other brain regions in HD in vivo models to see if the regional specificity of neurodegeneration is shifted.

Targeting Gpr52 is effective in lowering Htt and remarkably rescues HD phenotypes in vitro and in vivo (Figure 5), confirming its therapeutic potential. Further efforts need to be made to confirm its activity in mammalian and/or large animal models and to screen for its antagonists, which are currently unavailable.

### Gpr52's effect is cAMP-dependent

While the possible link between cAMP and Htt has been suggested previously (Williams et al., 2008; Lin et al., 2013), those studies focus on exogenously expressed or transgenic Htt aggregates or fragments, and show inconsistent results. Our data confirm that the sustained lowering of cAMP is required for GPCR-mediated lowering of endogenous soluble full-length Htt through PKA-independent mechanisms. It is intriguing why other striatal GPCRs, such as dopamine receptors, were not identified as Htt modulators by our screenings. There are several major possibilities besides a random missing of these targets. First, in the knock-down screen that we performed, the GPCRs need to have a certain level of constitutive basal activity in order to be identified as hits. Second, a sustained change of cAMP is required to modulate Htt levels (Figure 3D), and this may not be achieved by knocking-down many of the GPCRs in absence of agonists. Gpr52 may have a relatively high endogenous activity in the striatal cells, enabling it to provide a significant contribution to the endogenous cAMP levels. Alternatively, the endogenous Gpr52 ligand might be present at a certain level under endogenous conditions, and ligand is predicted to be fatty acids (Kakarala and Jamil, 2014). Finally, cAMP may only be a required part of the mechanism, while other signaling pathway or the receptor itself may contribute as well.

### The intronic gene and its host: *Gpr52* and *Rabgap1l*

*Gpr52* is located in the intron region of *Rabgap1l* gene in the mammalian genome, and the two genes are functionally linked in regulating Htt levels. This functional link is validated not only by the epistatic effects of Rabgap1l on Gpr52 mediated modulation (Figure 4A), but also confirmed by the involvement of Rab39B (Figure 4D), the substrate of Rabgap1l (Itoh et al., 2006). To our knowledge, this is so far the only pair of intronic and host genes showing functional links. The shared genomic locus of *Gpr52* and *Rabgapl1* may facilitate their balance in modulating Htt levels via co-regulated expression in certain cell types. For example, they may share the same enhancer regions or transcription factors in striatal cells. Alternatively, *Gpr52* mRNA might be cleaved and processed from the intron of *Rabgap1l* pre-RNA, possibly in a similar way as the biogenesis of intronic lncRNAs that have been recently identified (Yin et al., 2012; Zhang et al., 2013). These possibilities could be potentially tested by reconstitution of the relevant genome regions with reporters in a different system, and are beyond the scope of this study.

### Conclusion

In summary, we have identified Gpr52 as a striatal-enriched GPCR that stabilizes Htt in vitro and in vivo. It may contribute to the selective neurodegeneration and serve as a potential target for HD drug discovery. Our finding also implies that the selective vulnerability of striatal neurons may be contributed by striatal-enriched modulator of Htt levels. Identifying these modulators opens new therapeutic avenues for HD.

## Materials and methods

### Plasmid constructs

The Rap1-V12 (constitutively active Rap1), Rap1-N17 (dominant negative Rap1), Rap2-V12 (constitutively active Rap2) and Rap1-N17 (dominant negative Rap2) are kind gifts from Dr Daniel Pak. Their sequences and expression were further verified. All Rap mutants are in the mammalian expression vector pGW1 with the addition of an N-terminal HA (influenza hemagglutinin) epitope tag as previously described (Fu et al., 2007). The human GPR52 cDNA was synthesized in vitro and cloned into pDEST Gateway Vectors for transfections (Life Technologies, Grand Island, NY). The Rab39B cDNA was amplified from human brain first-strand cDNA library (Clonetech, Mountain View, CA cat no.S1199) and cloned into Hind III/Xho I sites of pcDNA3.0 (Life Technologies) with a FLAG-tag fused to C-teminus. The constitutively active forms (Q68L) were generated by point mutagenesis and verified by sequencing Rab39B.

### Cell culture and cell line generation

The mouse striatal cells (STHdh) were obtained from Coriell Cell Repositories (Camden, NJ). STHdh cells were cultured in DMEM (Life Technologies, cat. no. 11965) with 10% (vol/vol) FBS (Life Technologies, cat. no. 10082–147). For the generation of Huntington's disease iPSC lines, patient fibroblasts obtained from Coriell Cell Repositories (Q70) or from an HD patient (Q47) and his healthy sibling (WT, Q19) from one Mongolian family were transduced with the retroviral STEMCCA polycistronic reprogramming system (Millipore, Billarica, MA). The iPS lines were confirmed positive for Tra-1-81, Tra-1-60, SSEA-4 and Nanog by immunofluorescence and flow-cytometry and all four vector-encoded transgenes were found to be silenced. The study was approved by the ethic community of IBS at Fudan University (No.28). Verbal and written consent was obtained from all patients. iPSCs were maintained on a feeder layer of irradiated mouse embryonic fibroblasts (MEFs). The neuronal differentiation was performed as previously described (Ma et al., 2012). In briefly, iPSCs were differentiated to Pax6-expressing primitive neuroepithelia (NE) for 10–12 days in a neural induction medium. Sonic hedgehog (SHH, 200 ng/ml) was added at days 10–25 to induce ventral progenitors. For neuronal differentiation, neural progenitor clusters were dissociated and placed onto poly-ornithine/laminin-coated coverslips at day 26 in Neurobasal medium, with a set of trophic factors, including brain derived neurotrophic factor (BDNF, 20 ng/ml, Peprotech, Rocky Hill, NJ, cat. no. 450–02), glial-derived neurotrophic factor (GDNF, 10 ng/ml, Peprotech, cat. no. 450–10), insulin-like growth factor 1 (IGF1, 10 ng/ml, Peprotech, cat. no. 100–11) and Vitamin C (Sigma, St. Louis, MO cat. no. D-0260, 200 ng/ml). Primary mice cortex and striatum neurons were obtained from P0 pups, and tissues were digested with papain (Worthington Biochemical Corporation, Lakewood, NJ, cat. no. LS003119, 12 units/ml) and DNaseI (Roche Life Science, Indianapolis, IN, cat. no. 10104159001, 1 mg/ml) for 30 min with occasional mixing; digestion was stopped with 10% serum. Tissues were then triturated and plated in the Neurobasal @ medium (Life Technologies, cat. no. 21103–049) supplemented with 2% B27 (Life Technologies, cat. no. 17504044), 0.5× Pen/Strep (Life Technologies, cat. no. 15070–063) and 2 mM Glutamax (Life Technologies, cat. no. 35050–061) for striatal neurons (validated by Darpp32 staining), or 1% N2 (Life Technologies, cat. no. 17502–048), 2% B27, 0.5× Pen/Strep (Life Technologies, cat. no. 15140) and 2 mM Glutamax (Life Technologies, cat. no. 35050) for cortical neurons. All the cells were maintained at 37°C incubator with 5% CO_2_, except STHdh cells, which were maintained at 33°C with 5% CO_2_. All the cells were tested for mycoplasma contamination, but they have not been authenticated by STR profiling.

### Mouse models

The generation and characterization of the Hdh140Q knock-in mice have been previously described (Menalled et al., 2003). The Gpr52 knock-out was generated by Cyagen Biosciences Inc. (Guangzhou, China) using TALEN technology. A pair of TALEN constructs for Gpr52 knockout were cloned into a mammalian expression vector pCMV-TALEN and capped, polyA-tailed mRNA for injection were produced using the Ambion mMessage mMachine kit. The knockout mice were produced by microinjecting TALEN mRNAs into fertilized eggs from C57BL/6 strain. The knockout allele has been sequence validated to have eight missing base pairs (GAATGTGT, 57–64 of the ORF), causing a frameshift and an early stop (sequencing primers, forward: 5′-agccaaagctgcaaactccct-3′; reverse: 5′-gaaccaagcaggtaactccaacg-3′). The mRNA transcribed from targeted allele with frameshift undergoes nonsense-mediated decay. The mice were back-crossed to the wild-type background for five generations before crossing to the HD mice (with the same genetic background) or performing other experiments. The mouse experiments were carried out following the general guidelines published by the Association for Assessment and Accreditation of Laboratory Animal Care. The Animal Care and Use Committee of the School of Medicine at Fudan University approved the protocol used in animal experiments (Approval #20140904). For protein extraction from the mouse brain, the brains were collected and the striata and cortices were acutely dissected.

### cDNA and siRNA transfection

The siRNAs were reversely transfected into the STHdh cells with Lipofectamine 2000 (Life Technologies, cat. no. 11668) and into the iPSC-derived neurons with Lipofectamine RNAiMAX (Life Technologies, cat. no. 13778) according to the manufacturer's protocol. The cDNA were transfected with Lipofectamine 3000 (Life Technologies, cat. no. L3000) according to the manufacturer's protocol. Cells were collected 3 days after siRNA transfection or 2 days after cDNA transfection for western-blot, HTRF or immunofluorescence. For experiments with both cDNA and siRNA transfections, the cDNA transfection was performed 1 day after the siRNA transfection with culture medium replacement, and the cells were collect 2 days after the cDNA transfection. siRNA target sequences: mouse Gpr52_si1: GGATCGATATCTTGCAATA, Gpr52_si2: GGATAACTAGCGTGTTTTA, Gpr52_si3: GTGGATCGATATCTTGCAATA; human hGpr52_si1: TGTCGCTTGAGAATTTGCATTATTT, hGpr52_si2: GACAATCCAACTCTGTCCTTCTTAA; mouse Htt siRNA: ACCGTGTGAATCATTGTCTAA (Hdh5), CTCATTGTGAATCACATTCAA (B01), CTGGTTGGTATTCTTCTAGAA (C01); human Htt siRNA (HTT3): CAGGTTTATGAACTGACGTTA; mouse Rabgap1l siRNAs: mouse Rabgap1l siRNA1: GCAGUGAAGUGGAGGCUUUTT, Rabgap1l siRNA2: GCUAUGAUGGGAGAGCUUA; human Rabgap1l siRNA: GCUAUGAUGGGAGAGCUUA (target both human and mouse).

### Compound treatment

For compound treatment, the cells were plated at the same density as the siRNA transfection, and the compounds were diluted in OPTI-MEM to 10× concentrations and added 1–2 days later. The cells were then collected 1–2 days later for further analysis. Compound ordering information is as follows: forskolin (Sigma, cat. no. F6886), Rp-cAMP (Sigma, cat. no. A165), 8-pCPT-2O′-Me-cAMP (Sigma, cat. no. C8988), 6-Bnz-cAMP (Sigma, cat. no. B4560), 8-Br-cAMP (Sigma, cat. no. B5386), H-89 (Sigma, cat. no. B1427), Reserpine (Selleck, Houston, TX, cat. no. S1601), Bafilomycin A (Sigma, cat. no. B1793), Epoxomicin (Cayman Chemical, Ann Arbor, MI, cat. no. BU4061T), MG132 (Sigma, cat. no. M7449), chloroquine (Sigma, cat. no. C6628), Ammonium chloride (Sigma, cat. no. A9434).

### cAMP and PKA activity measurement

The assays utilize the cAMP-Glo Assay kit purchased from Promega (Fitchburg, WI, cat. no. V1501). For cAMP measurement in cells, chemicals were added 24 hr before measurement. On the day of measurement, change medium containing compound into PBS with phosphodiesterase inhibitors, and incubate 2 hr and then the procedures were performed following manufacture's instruction. Purified cAMP provided by the kit was diluted into different concentrations to plot the standard curve. The slope of the standard curve was utilized to determine the cAMP concentration change per unit change of the signal. For PKA activity measurement, compounds diluted in Opti-MEM at indicated concentrations were directly added into 384 well plates, and then the experiments were performed following manufacture's instruction.

### Protein extraction and western-blots

For standard western-blots, the cell pellets were collected and lysed on ice for 30 min in PBS + 1% (vol/vol) Triton X-100 + 1× Complete Protease Inhibitor (Roche Diagnostics, Indianapolis, IN, cat. no. 04693116001), sonicated for 10 s, and spun at >20,000×*g* at 4°C for 10 min. The supernatants were then loaded and transferred onto nitrocellulose membranes for western blots. For fractionation experiments that extract Htt proteins at different fractions, the cell lysates are fractionated into P1, P2 and S2 fractions according to the previously described protocol (Kegel et al., 2005). Briefly, cell homogenates made in the homogenate buffer (10 mm HEPES, pH 7.4, 250 mm sucrose, 1 mm EDTA plus 1× Complete Protease Inhibitor) were centrifuged at 2000×*g* to obtain the crude nuclear pellet (P1) and the postnuclear supernatant (S1). S1 was then centrifuged at 100,000×*g* to obtain the membrane pellet (P2) and the cytosolic fraction (S2). The P1 and P2 pellets were then washed with the homogenate buffer and resuspended in 1× PBS buffer with 2% SDS by sonication on ice for 10 s. Equal amount (10–20 μg of total proteins) of each fraction was loaded in each lane for western-blots. For detection of the Gpr52 protein, the cells are lysed in 2% Fos-choline-14 in 1× PBS to extract the membrane proteins. For mouse brain samples, all mice striatum and cortex tissues protein extracts were obtained in the following ice-cold extraction buffer: 50mM Tris.HCl pH 7.4, 250mM NaCl, 5mM EDTA.Na_2_, 1% Triton-100 with 1× Complete Protease Inhibitor, and then homogenized by electric homogenizer. The lysates were centrifuged at 20,000 g at 4°C for 20 min, and the supernatants were used for western-blots. The HTT antibodies 2B7 (Weiss et al., 2009) (1:1000), ab1 (Sapp et al., 2012) (1:3000) and MW1 (Ko et al., 2001) (1:1000) have been described previously. The antibody S830 (Sathasivam et al., 2001) (1:10000) is a kind gift from Dr Gillian Bates. Commercially purchased antibodies include HTT antibody 2166 (Millipore, cat. no. MAB2166, 1:1000), 3B5H10 (Sigma, cat. no. P1874, 1:1000), 2050 (AbD Serotec, Raleigh, NC, cat. no. MCA2050) and 2051 (AbD Serotec, cat. no. MCA2051), anti-â-tubulin (Abcam, Cambridge, MA, #ab6046, 1:5000), anti-Calnexin (Stressgen, San Diego, CA, cat. no. pAb-ADI-SPA-860, 1:2000), anti-Gpr52 (GeneTex, Irvine, CA, cat. no. GTX108123, 1:500), anti-Ataxin3 (Millipore, cat. no. MAB5360) and anti-HA (Santa Cruz Biotechnology, Santa Cruz, CA, cat. no. sc-805, 1:500). Note that anti-Gpr52 antibody only detects human Gpr52 but not the mouse Gpr52. We failed to generate anti-mouse Gpr52 antibody or mouse Gpr52 cDNA plasmids. Similar technical difficulties have been also reported by others (Komatsu et al., 2014). All secondary antibodies were used at 1:5000. For all the representative western-blots shown in the figures, three or more biological repeats have been performed showing consistent results.

### Homogeneous time resolved fluorescence (HTRF) assay

The HTRF assays were performed similarly to those previously described (Lu et al., 2013; Liang et al., 2014). For all the samples, the protein concentration (by BCA, Life Technologies, cat. no. 23225) and/or the DNA content (by Picogreen, Life Technologies, cat. no. P7589, for lyse-in-well experiments) were measured to correct the loadings. Different protein concentrations or cell numbers per well were tested to ensure that the signals were in the linear range. Background corrections were performed by subtracting the background signals from blank samples.

### Immunofluorescence

Coverslip cultures were fixed in 4% paraformaldehyde for 15–20 min, washed with 1× PBS 10 min three times and incubated in a blocking buffer (10% donkey serum and 0.2% triton X-100 in PBS) for 60 min and then incubated with primary antibodies: Tuj1 (Covance, Princeton, NJ, USA, cat. no. 14971502, 1:5000), GABA (Sigma, cat. no. A0310, 1:200), DARPP32 (Millipore, cat. no. AB1656, 1:1000), Htt antibodies 2B7 (Weiss et al., 2009) (1:200), 2050 or 2051 (Bio-rad, MCA2050 or MCA2051, 1:200), Rab22A antibody (abcam, cat. no. ab137093), or Rab39B antibody (abcam, cat. no. ab154826) overnight at 4°C. Fluorescence conjugated secondary antibodies were used to reveal the binding of primary antibodies (Life Technology, cat. no. A21206, A31572, A21202, A21203, 1:1000) and nuclei were stained with DAPI (Sigma, cat. no. D9542, 1:1000). Images were captured by Leica TCS SP8 confocol system. Co-localization was quantified using the Pearson's correlation coefficient and the overlap coefficient, which were calculated with Image-pro Plus software. Data represent the mean with standard deviation. One-way ANOVA experiments were performed to judge the statistical significance.

### qPCR

mRNA levels were determined by qPCR. RNA from siRNA-transfected or compound treated cells was extracted using RNAprep Pure Cell/Bacteria Kit (Tiangen, Beijing, China, cat. no. DP430). Random-primed cDNA was obtained by reverse transcription using the FastQuant RT Kit (Tiangen, cat. no. KR106). DNase I was added to break down the genomic DNA. qPCR was then performed using SYBR Green Realtime PCR Master Mix (Toyobo, Osaka, Japan, cat. no. QPK-201). qPCR Primers used were as follows: Gpr52 forward: TTGCTTTATTGTTTGTTTACTTTATGC, Gpr52 reverse: GTGAAAGTAAGTGAAGCAGACAACC; Rabgap1l forward: GGAACTGGCACAGACCAAAC, Rabgap1l reverse: GCTCCTCTCTGATGCTCAAGTT; Hprt forward: GTCAACGGGGGACATAAAAG, Hprt reverse: CAACAATCAAGACATTCTTTCCA; Htt forward: CTGCACGGCATCCTCTATGT, Htt reverse: TGTTCACGCAGTGGGCTATT.

All the primers were tested with standard curve, amplification efficiency was between 95%–105%, and the R^2^ for linear relationship is >0.999. No reverse-transcriptase controls were used to ensure the specificity of the signals.

### Neuronal loss assays

The patient iPS-derived neurons exhibit HD-dependent phenotypes including elevated caspase-3 signals and neuronal loss upon BDNF removal. Similarly, the STHdh cells exhibit HD-dependent caspase 3 and/or 7 activity upon stress, such as serum removal. Briefly, patient and wild type iPS-derived neurons were cultured in NIM (1% N_2_ in DMEM:F12) for further 48 hr after transfected with siRNAs for 4 days. These phenotypes could be detected by the caspase activity assay as well as the neuronal loss assay. For the caspase activity assay, the NucView 488 caspase-3 dye (Biotium, Hayward, CA, cat. no. 30029) was used for the caspase activity detection as based on manufacturer's protocols. The images of the caspase-3 dye and DAPI treated live cells were taken by Nikon ECLIPSE TE2000-S microscope. For the neuronal loss experiments, the Tuj1 confluence and shape were analyzed by ImageJ and the sholl analysis-plug in of ImageJ (Sholl, 1953). The images were analyzed blindly.

### *Drosophila* motor performance tests

Experiments were performed using 15 age-matched virgin females. We placed the flies in an empty vial and tapped them down. The percentage of flies that climbed past a 9-cm-high line after 18 s was recorded, and two replicates were tested in parallel for each genotype. The mean and S.E.M. of 10 observations is plotted for each day and data analyzed by ANOVA followed by Dunnett's post hoc test. Blinding was used both for carrying out the experiment and for analyzing the data. The nervous system driver line *elav-GAL(c155)* as well as the classical loss-of-function alleles DopEcR^MI02790^ and GapcenA^f01044^ were obtained from the Bloomington *Drosophila* Stock Center at University of Indiana (http://flystocks.bio.indiana.edu/). The inducible shRNA lines DopEcR^KK103494^ and GAPcenA^KK103588^ were obtained from the Vienna Drosophila Resource Center (http://stockcenter.vdrc.at/control/main/). NT-Htt128Q flies express an N-terminal human Htt fragments comprising exons 1–4 (first 336 amino acids including 128Q) and have been previously described (Al-Ramahi et al., 2006). FL-Htt200Q flies express the full length human Htt protein with 200Q and exhibit similar motor defects (Video 1).

### Statistical analysis

Statistical comparisons were conducted by the two-tailed unpaired Mann–Whitney U-test for comparing the average of each sample in the bar graphs. For comparison of sample averages with different doses of Gpr52 cDNA transfected vs the single control (mock) (Figure 2C), one way ANOVA tests were performed followed by Dunnett's *post hoc* test. For comparisons between different groups over different time points, two-way ANOVA has been utilized (Figure 3C–D). Significance was established at p < 0.05. In all graphs, error bars, S.E.M. The biological replicate numbers are indicated on top of each bar and/or as the n numbers in the legends. The statistical powers for all analyses were calculated and confirmed to be >80%. For all the cellular assays, the cells were evenly suspended and then randomly allocated in each well tested. For the animal experiments, the littermates were allocated into each group based on their genotypes and thus randomization does not apply. The key experiments including detecting Htt level changes upon Gpr52 knock-down or knockout, the co-localization analysis, and the cellular phenotypic rescue analyses were confirmed by blind testing in which different investigators prepared/labeled the samples and the analyses were carried out without information exchange before obtaining the results. The key observations including the lowering of Htt, the epistasis between Gpr52 and Rabgap1l and the phenotypic rescue have been replicated by multiple independent investigators. Data were excluded when there were clear indications of artifact or experimental failures, such as contamination, transfection/infection failure, etc.

### Virus packaging

Lentiviral particles for infection were produced in HEK293T cells transfected with the pLKO-Gpr52-shRNA or scrambled constructs with the three plasmid packaging mix (pLP1, pLP2 and pLPVSV-G). After 48 hr, the medium was collected, filtered through a 0.45-µm membrane and ultracentrifuged at 20,000 rpm for 90 min. The viral pellets were resuspended in sterile PBS with 1% BSA and stored at −80°C. Viral titers were determined using Abm's qPCR Lentivirus Titration Kit (Abm, Richmond, Canada, cat. no. LV900). The Gpr52 shRNA target sequences are: Gpr52_sh1: CTCCGCTGTTACACCATTATA; Gpr52_sh2: GTGGATCGATATCTTGCAATA. The viruses were applied at M.O.I = 3 for the primary neurons, and the knock-down of Gpr52 was confirmed by qPCR.
